# Role for RIP1 in mediating necroptosis in experimental intracerebral hemorrhage model both *in vivo* and *in vitro*

**DOI:** 10.1038/cddis.2017.58

**Published:** 2017-03-02

**Authors:** Haitao Shen, Chenglin Liu, Dongping Zhang, Xiyang Yao, Kai Zhang, Haiying Li, Gang Chen

**Affiliations:** 1 Department of Neurosurgery and Brain and Nerve Research Laboratory, The First Affiliated Hospital of Soochow University, Suzhou, Jiangsu Province, China

## Abstract

Cell death is a hallmark of second brain injury after intracerebral hemorrhage (ICH); however, the mechanism still has not been fully illustrated. In this study, we explored whether necroptosis, a type of regulated necrosis, has an essential role in brain injury after ICH. We found that inhibiting receptor-interacting protein 1 (RIP1) – a core element of the necroptotic pathway – by a specific chemical inhibitor or genetic knockdown attenuated brain injury in a rat model of ICH. Furthermore, necroptosis of cultured neurons could be induced by conditioned medium from microglia stimulated with oxygen hemoglobin, and this effect could be inhibited by TNF-*α* inhibitor, indicating that TNF-*α* secreted from activated microglia is an important factor in inducing necroptosis of neurons. Undoubtedly, overexpression of RIP1 increased conditioned medium-induced necroptosis *in vitro*, but this effect was partially diminished in mutation of serine kinase phosphorylation site of RIP1, showing that phosphorylation of RIP1 is the essential molecular mechanism of necroptosis, which was activated in the *in vitro* model of ICH. Collectively, our investigation identified that necroptosis is an important mechanism of cell death in brain injury after ICH, and inhibition of necroptosis may be a potential therapeutic intervention of ICH.

Intracerebral hemorrhage (ICH) is the second largest type of stroke, accounting for ~15% of all patients with stroke.^1, 2^ ICH is associated with fast progression, high mortality and high morbidity. It has been reported that, with mortality rates close to 40% in 1 month, patients have legacy paralysis, aphasia and other severe disabilities after ICH. The incidence of ICH also increased significantly with the increase of population age.^3^ Primary brain injury after ICH is due to hematoma mass effect and mechanical damage to adjacent brain tissues,^4^ and the secondary brain injury is a key reason to cause nerve function damage in patients with ICH.^5, 6^ In secondary brain injury after ICH, pathological changes, including cell death, cerebral edema and blood–brain barrier (BBB) damage, occur in brain tissues surrounding hematoma.^7^ Mechanism of secondary brain injury after ICH includes excitatory amino-acid toxicity, inflammatory response, expression of proteolytic enzyme and the toxic effects of hematoma release product.^5, 7^ Cell death is an important factor in secondary brain injury after ICH.

In recent years, necrosis as another form of cell death causes more attention. The definition of necrosis is based on its pathological morphological characteristics; it is a passive cell death mostly caused by overwhelming stress such as dramatic changes in temperature or pH. Necrotic cells rapidly lose cell membrane integrity, and cell membrane swelling and mitochondrial dysfunction occur. The membrane rupture caused a large number of cytoplasmic components to leak from the cell and then induced inflammation in the surrounding tissues.^8^ Previous studies suggested that necrosis is an occasional and irregular event and is difficult to study.^9^ However, Yuan and co-workers^10^ reported, for the first time, necroptosis as a form of necrosis that can be regulated.^10^ Necroptosis has similar morphological characteristics (including early membrane integrity, cell and intracellular organelle swelling) with necrosis, but it is caspase-independent programmed cell death.^11^

Recent studies have shown that death receptors on the cell membrane mediated different pathways of cell death depending on the state of cell or local physiological or pathophysiological microenvironment; for example, in the event of TNF-*α*-induced cell death, TNF-*α* and its receptors firstly formed a trimer, and then recruited proteins containing the death domain (DD) formed a complex, which activated the downstream apoptotic pathway. However, if caspase activity was inhibited, the cells would go to the necroptotic pathway. RIP1 was recruited to TNFR1 through its DD, which bound directly to the DD of TNFR1, and it was activated by multiple forms of ubiquitination. The activation of RIP1 led to the recruitment of RIP3, MLKL and caspase-8, forming a complex called 'necrosome' involved in necroptotic pathway. Thus, RIP1 played the role of adaptor in mediating necroptosis. Activation of RIP3 activated three key enzymes of cell metabolic pathways – glycogen phosphorylase, glutamine synthetase, and glutamate dehydrogenase – which caused overproduction of reactive oxygen species (ROS). ROS caused DNA damage, damage to mitochondrial membrane permeability and lysosome damage, eventually leading to cell death.^11, 12^

Previous studies have confirmed that microglia was quickly activated in brain tissues after ICH, and it secreted a large number of inflammatory factors, including the TNF-*α*, which can induce necroptosis.^13^ Under the stimulation of TNF-*α*, a part of the brain cells undergo apoptosis and a part may undergo necroptosis in brain tissues after ICH. Necrotic cell death is common in a wide variety of pathological conditions, including stroke.^14^ Compared with numerous investigations on the mechanisms of apoptotic cell death, fewer studies have explored necrotic cell death in ICH. Here we studied the role of necroptosis, a type of regulated necrosis, in ICH.

## Results

### Necroptosis was activated in brain tissues after ICH

**Figure 1:**
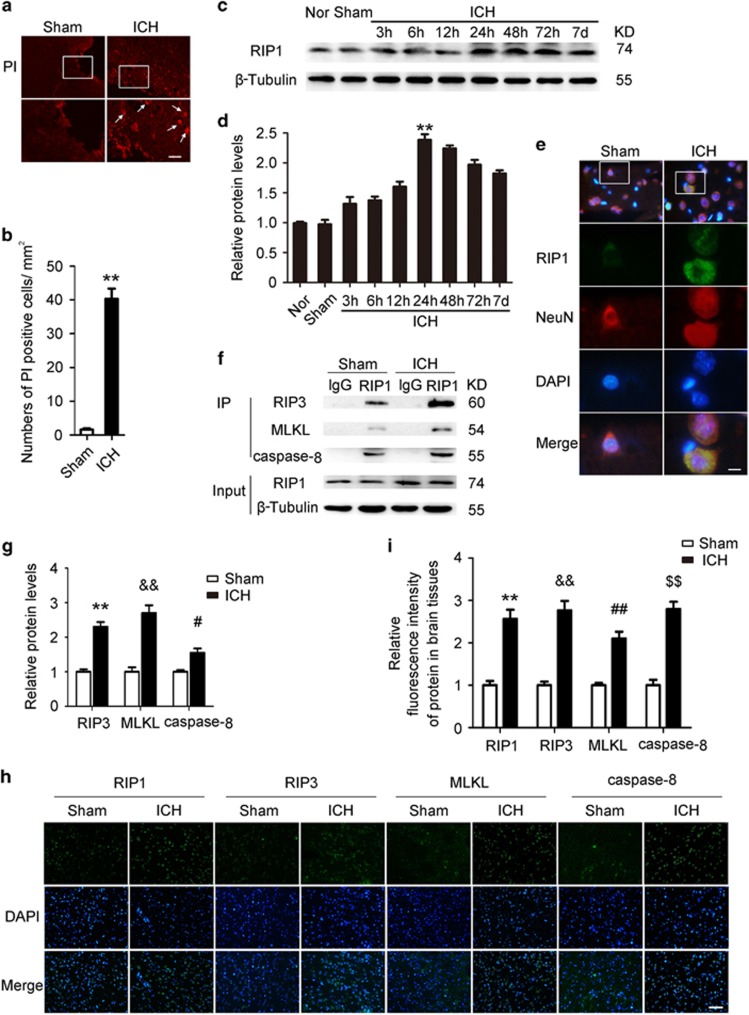
Indicators of necroptosis were discovered in brain tissues after ICH. (**a**) The necroptosis of cells in brain tissues was detected by PI labeling. As shown, compared with the Sham group, considerable PI+ cells were detected in frozen sections of brain tissues in rats at 24 h after ICH. Arrows point to PI+ cells. Scale bar=50 *μ*m. (**b**) Related with (**a**), it revealed relative levels of PI+ cells, **P<0.0001 *versus* Sham group, unpaired *t*-test, *n*=6. (**c**) The results of western blot suggested that the expression levels of RIP1, a major regulator for necroptosis, were obviously upregulated, and it reached peak at 24 h in brain tissues after ICH. (**d**) Related with (**c**), quantitative analysis of expression levels of RIP1 in brain tissues within 1 week after ICH. **P=0.0003 *versus* Sham group, unpaired *t*-test, *n*=3. (**e**) Double immunofluorescence (IF) analysis was performed with antibodies for RIP1 (green) and NeuN (red). Nuclei were fluorescently labeled with DAPI (4',6-diamidino-2-phenylindole) (blue). Representative images of the Sham group and the ICH (24 h) group were shown. Scale bar=10 *μ*m. (**f**) The formation of necrosome was detected by immunoprecipitation (IP) by using anti-RIP1 antibody (rabbit immunoglobulin G (IgG) was also used as a negative control), and RIP3, MLKL and caspase-8 were detected by immunoblotting. The results suggested that increased interactions of RIP1 and RIP3, RIP1 and MLKL, and RIP1 and caspase-8 were observed in brain tissues at 24 h after ICH. Input, 5% of extract before IP. (**g**) Quantitative analysis of IP. **P=0.0011 *versus* Sham group; ^&&^*P*=0.0011 *versus* Sham group; ^#^*P*=0.0152 *versus* Sham group; all were unpaired *t*-test, *n*=3. Besides, results of IF (**h**) showed that expressions of these four proteins, which constituted necrosome, were increased significantly in brain tissues at 24 h after ICH than those in the Sham group. Scale bar=100 *μ*m. Related statistic of data was revealed in (**i**). **P<0.0001 *versus* Sham group; ^&&^*P*<0.0001 *versus* Sham group; ^##^*P*<0.0001 *versus* Sham group; ^$$^*P*<0.0001 *versus* Sham group; all were unpaired *t*-test, *n*=6. All data are expressed as means±S.E.M., mean value for the Sham group was normalized to 1.0

To explore the involvement of necroptosis in brain tissues after ICH, we detected propidium iodide-positive (PI+) cells in frozen brain sections at 24 h after ICH. Notably, compared with that in the Sham group, the PI+ cells were remarkably increased in brain tissues surrounding hematomas after ICH (*P*<0.0001, *n*=6; Figures 1a and b). We also determined the expression of RIP1, which is a major regulator for necroptosis in brain tissues; the results of western blot suggested that the expression levels of RIP1 were progressively upregulated and peaked at 24 h after ICH (*P*=0.0003 *versus* Sham group, *n*=3; Figures 1c and d). The results of immunofluorescent staining also showed that the expression level of RIP1 in neurons was remarkably increased at 24 h after ICH (Figure 1e).

We further evaluated the formation of necrosome, which is an important hallmark of activation of necroptosis in brain tissues after ICH; anti-RIP1 antibody was used by immunoprecipitation (IP), and RIP3, MLKL and caspase-8 were detected by immunoblotting. Increased interactions of RIP1 and RIP3, RIP1 and MLKL, and RIP1 and caspase-8 were observed in brain tissues after ICH (all *P*<0.01 *versus* Sham group, *n*=3, Figures 1f and g). It suggested that the formation of necrosome was significantly increased in brain tissues after ICH than that in the Sham group. The results of immunofluorescence also showed that expressions of these four proteins were significantly upregulated after ICH than that in the Sham group (all *P*<0.01 *versus* Sham group, *n*=6, Figures 1h and i). These results indicated that necroptosis was activated in brain tissues after ICH.

### Inhibitor of necroptosis and apoptosis differentially attenuates brain injury after ICH

**Figure 2:**
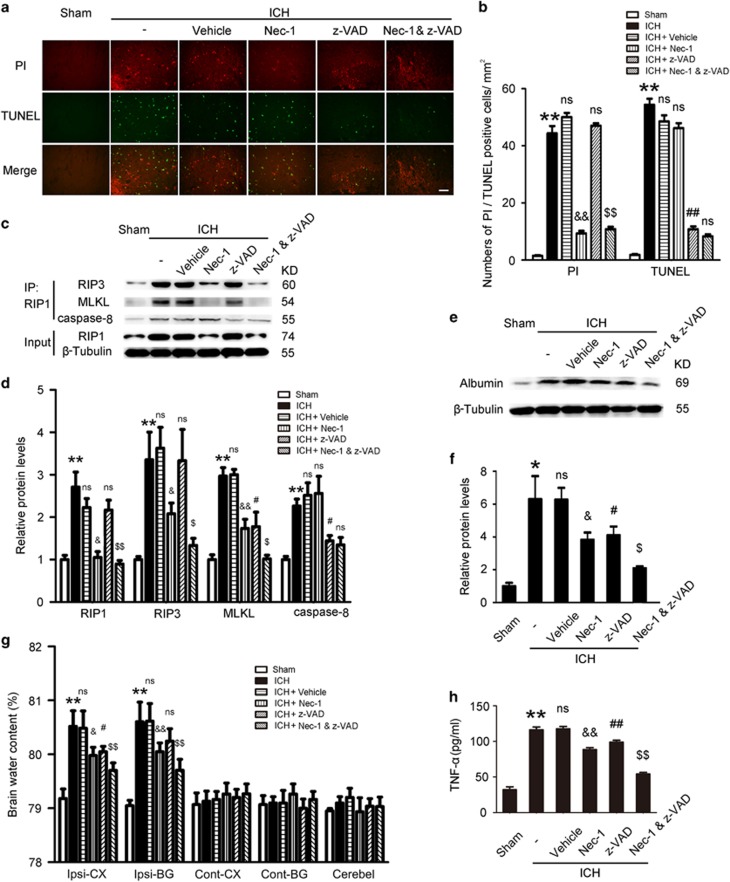
Different effects of Nec-1 and z-VAD on brain injury after ICH. Nec-1 (the specific inhibitor of necroptosis) and z-VAD (a caspase inhibitor) were used to explore whether necroptosis contributes to brain injury after ICH. (**a**) The necroptosis in cells were detected by PI staining and apoptosis by terminal deoxynucleotidyl transferase dUTP nick-end labeling (TUNEL) staining. Nec-1 reduced the PI+ cells, whereas it had no significant effects on TUNEL+ cells (apoptosis), and z-VAD only downregulated the TUNEL+ cells. Additionally, combination of Nec-1 and z-VAD obviously both reduced the PI+ cells and TUNEL+ cells. Scale bar=100 *μ*m. (**b**) Corresponding bar graph revealed relative levels of PI/TUNEL+ cells. **P<0.0001 (both in PI and TUNEL) *versus* Sham group; NS, not significant difference (*P*=0.0909 in PI and *P*=0.0857 in TUNEL) *versus* ICH group; ^&&^*P*<0.0001 in PI and NS (*P*=0.4233) in TUNEL *versus* vehicle group; NS (*P*=0.4233) in PI and ^##^*P*<0.0001 in TUNEL *versus* vehicle group; ^$$^*P*<0.0001 in PI and NS (*P*=0.0663) in TUNEL *versus* z-VAD group; all were unpaired *t*-test, *n*=6. (**c**) IP showed that Nec-1 reduced interactions of RIP1 and RIP3, RIP1 and MLKL, and RIP1 and caspase-8, indicating that the formation of necrosome was inhibited. Input, 5% of extract before IP. (**d**) Corresponding bar graph revealed relative levels of association. **P=0.0097 in RIP1, **P=0.0099 in RIP3, **P=0.0010 in MLKL and **P=0.0022 in caspase-8 *versus* Sham group; NS, not significant difference (*P*=0.3077 in RIP1, *P*=0.7942 in RIP3, *P*=0.9047 in MLKL and *P*=0.4930 in caspase-8) *versus* ICH group; ^&^*P*=0.0102 in RIP1, ^&^*P*=0.0493 in RIP3, ^&&^*P*=0.0074 in MLKL and NS (*P*=0.9349) in caspase-8 *versus* vehicle group; NS (*P*=0.8658) in RIP1, NS (*P*=0.6857) in RIP3, ^#^*P*=0.0205 in MLKL and ^#^*P*=0.0281 in caspase-8 *versus* vehicle group; ^$$^*P*=0.0065 in RIP1, ^$^*P*=0.0499 in RIP3, ^$^*P*=0.0403 in MLKL and NS (*P*=0.6746) in caspase-8 *versus* z-VAD group; all were unpaired *t*-test, *n*=3. (**e**) Expression of albumin, which is regarded as the index of BBB injury, was increased after ICH, whereas it could be significantly decreased when treated with Nec-1 and/or z-VAD. (**f**) Corresponding bar graph revealed relative levels of albumin. *P=0.0200 *versus* Sham group; NS, not significant difference (*P*=0.9779) *versus* ICH group; ^&^*P*=0.0461 *versus* vehicle group; ^#^*P*=0.0496 *versus* vehicle group; ^$^*P*=0.0126 *versus* z-VAD group; all were unpaired *t*-test, *n*=6. (**g**) Compared with ICH group, the brain water content was partially attenuated in Nec-1 and/or z-VAD group, **P<0.0001 (both in Ipsi-CX and Ipsi-BG) *versus* Sham group; NS, not significant difference (*P*=0.9022 in Ipsi-CX and *P*=0.9660 in Ipsi-BG) *versus* ICH group; ^&^*P*=0.0109 in Ipsi-CX and ^&&^*P*=0.0077 in Ipsi-BG *versus* vehicle group; ^#^*P*=0.0162 in Ipsi-CX and NS (*P*=0.0727) in Ipsi-BG *versus* vehicle group; ^$$^*P*=0.0015 in Ipsi-CX and ^$$^*P*=0.0039 in Ipsi-BG *versus* z-VAD group; all were unpaired t-test, *n*=6. (**h**) The levels of TNF-*α* in the CSF were measured by ELISA and reduced by Nec-1 treatment. **P<0.0001 *versus* Sham group, NS, not significant difference (*P*=0.7897) *versus* ICH group; ^&&^*P*<0.0001 *versus* vehicle group, ^##^*P*=0.0008 *versus* vehicle group, ^$$^*P*<0.0001 *versus* z-VAD group; all were unpaired *t*-test, *n*=6. All data are expressed as means±S.E.M. and mean value for Sham group was normalized to 1.0.

To determine whether necroptosis contributes to brain injury after ICH, the specific inhibitor of RIP1, necrostatin-1 (Nec-1), was used, and z-VAD, a caspase inhibitor, which can inhibit apoptosis, was also used as a positive control in a rat model of ICH. Nec-1 reduced the PI+ cells but had no significant effect in TUNEL+ cells, whereas z-VAD only downregulated the TUNEL+ cells, and did not affect the PI+ cells in brain tissues after ICH. The combination of z-VAD and Nec-1 both obviously reduced the PI+ cells and TUNEL+ cells (all *P*<0.01, *n*=6; Figures 2a and b). The results of IP also demonstrated that interactions of RIP1 and RIP3, RIP1 and MLKL, and RIP1 and caspase-8 were significantly inhibited by Nec-1 but not by z-VAD (all *P*<0.05, *n*=3; Figures 2c and d). These results indicated that necroptosis and apoptosis occurred in different cells at same times and were independent of each other in brain tissues after ICH.

BBB permeability was assessed using albumin extravasation, and the western blot was used to test the albumin level in brain tissues; the results also suggested that treatment with Nec-1 in ICH rats can improve albumin extravasation and BBB injury (*P*=0.0461, *n*=3; Figures 2e and f). Brain water content was calculated by the wet and dry weight method; the results showed that, compared with ICH group, the brain water content was significantly reduced in the ICH+Nec-1 group (*P*=0.0109 in Ipsi-CX and *P*=0.0077 in Ipsi-BG, *n*=6; Figure 2g). The neurological score of the ICH+Nec-1 group was significantly lower than that in the ICH group (*P*=0.0094, *n*=6; Table 1). The levels of TNF-*α* in the cerebrospinal fluid (CSF) were measured by ELISA; the results confirmed that the level of TNF-*α* was obviously reduced by Nec-1 treatment, indicating that Nec-1 inhibits inflammation in ICH rats (*P*<0.0001, *n*=6; Figure 2h). These results suggested that necroptosis contributes to brain injury after ICH, including neuronal dysfunction, brain water content, BBB permeability and inflammation.

### Blockade necroptosis by knockdown of RIP1 improves brain injury after ICH

**Figure 3:**
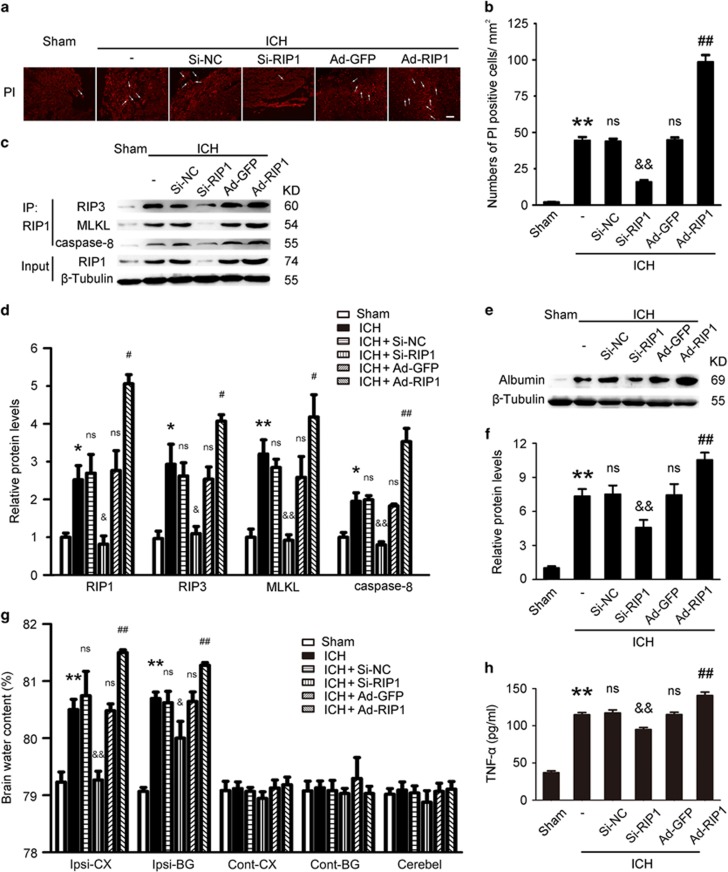
Knockdown of RIP1 reduced necroptosis in brain tissues after ICH. Knockdown and overexpression of RIP1 were used to study the role of RIP1-mediated necroptosis after ICH in rats. (**a**) PI+ cells decreased in the Si-RIP1 group (knockdown), whereas they increased in the Ad-RIP1 group (overexpression). Arrows point to PI+ cells. Scale bar=200 *μ*m. (**b**) Related with (**a**), it revealed relative levels of PI+ cells. **P<0.0001 *versus* Sham group; NS, not significant difference (*P*=0.8453) *versus* ICH group; ^&&^*P*<0.0001 *versus* Si-NC group; NS, not significant difference (*P*=0.9211) *versus* ICH group; ^##^*P*<0.0001 *versus* Ad-GFP group; all were unpaired t-test, *n*=6. (**c**) IP demonstrated that interactions of RIP1 and RIP3, RIP1 and MLKL, and RIP1 and caspase-8 were significantly inhibited in the RIP1-knockdown group, whereas they increased in the overexpression group. Input, 5% of extract before IP. Quantitative analysis of IP was shown in (**d**), *P=0.0177 in RIP1, *P=0.0261 in RIP3, **P=0.0072 in MLKL and *P=0.0209 in caspase-8 *versus* Sham group; NS, not significant difference (*P*=0.7939 in RIP1, *P*=0.6515 in RIP3, *P*=0.4640 in MLKL and *P*=0.8812 in caspase-8) *versus* ICH group; ^&^*P*=0.0254 in RIP1, ^&^*P*=0.0184 in RIP3, ^&&^*P*=0.0019 in MLKL and ^&&^*P*=0.0010 in caspase-8 *versus* Si-NC group; NS (*P*=0.7254) in RIP1, NS (*P*=0.5590) in RIP3, NS (*P*=0.3154) in MLKL and NS (*P*=0.6284) in caspase-8 *versus* ICH group; ^#^*P*=0.0164 in RIP1, ^#^*P*=0.0138 in RIP3, ^#^*P*=0.0367 in MLKL and ^##^*P*=0.0083 in caspase-8 *versus* Ad-GFP group; all were unpaired *t*-test, *n*=3. (**e**) Expression of albumin was elevated in the Ad-RIP1 group, whereas it was opposite in the Si-RIP1 group. (**f**) Bar graph related to (**e**). **P=0.0007 *versus* Sham group; NS, not significant difference (*P*=0.8780) *versus* ICH group; ^&^*P*=0.0493 *versus* Si-NC group; NS, not significant difference (*P*=0.9361) *versus* ICH group; ^#^*P*=0.0470 *versus* Ad-GFP group; all were unpaired *t*-test, *n*=3. (**g**) Brain water content decreased in the Si-RIP1 group, whereas it was opposite in the Ad-RIP1 group,**P<0.0001 (both in Ipsi-CX and Ipsi-BG) *versus* Sham group; NS, not significant difference (*P*=0.4041 in Ipsi-CX and *P*=0.5003 in Ipsi-BG) *versus* ICH group; ^&&^*P*<0.0001 in Ipsi-CX and ^&^*P*=0.0209 in Ipsi-BG *versus* Si-NC group; NS, not significant difference (*P*=0.5276 in Ipsi-CX and *P*=0.6015 in Ipsi-BG) *versus* ICH group; ^##^*P*<0.0001 (both in Ipsi-CX and Ipsi-BG) *versus* Ad-GFP group; all were unpaired *t*-test, *n*=6. (**h**) The levels of TNF-*α* in the CSF were reduced in RIP1-knockdown group, whereas the effect was diametric in the RIP1 overexpression group. **P<0.0001 *versus* Sham group; NS, not significant difference (*P*=0.5570) *versus* ICH group; ^&&^*P*=0.0002 *versus* Si-NC group; NS, not significant difference (*P*=0.9151) *versus* ICH group; ^##^*P*<0.0001 *versus* Ad-GFP group; all were unpaired t-test, *n*=6. All data are expressed as means±S.E.M., and mean values for Sham group were normalized to 1.0. Ad-GFP, adenovirus with GFP; Ad- RIP1, adenovirus with RIP1; Si-NC, Si-negative control.

To further define the role of RIP1-mediated necroptosis in neuronal dysfunction after ICH, we used knockdown by small interfering RNA (siRNA) interference and overexpression by recombinant adenoviruses transfection of RIP1 as treatment in rat model of ICH. Knockdown of RIP1 reduced the PI+ cells, but overexpression of RIP1 increased the PI+ cells in brain tissues after ICH (both *P*<0.0001, *n*=6; Figures 3a and b). The results of IP also demonstrated that interactions of RIP1 and RIP3, RIP1 and MLKL, and RIP1 and caspase-8 were significantly inhibited by knockdown of RIP1, but they were increased by overexpression of RIP1 (all *P*<0.05, *n*=3; Figures 3c and d). These results indicated that knockdown of RIP1 can effectively block necroptotic pathway.

The results of BBB permeability also suggested that treatment with RIP1 siRNA in ICH rats can inhibit albumin extravasation and BBB permeability (*P*=0.0493, *n*=3; Figures 3e and f). The results of brain water content declared that, compared with the ICH group, the brain water content was significantly reduced in the RIP1 siRNA group (*P*<0.0001 in Ipsi-CX and *P*=0.0209 in Ipsi-BG, *n*=6; Figure 3g). The neurological score of the ICH+Si-RIP1 group was significantly lower than that of the ICH group (*P*=0.0185, *n*=6; Table 1). The levels of TNF-*α* in the CSF were detected by ELISA; the results confirmed that the level of TNF-*α* was reduced by RIP1 siRNA treatment, indicating that downregulation of RIP1 could inhibit inflammation in brain tissues after ICH (*P*=0.0002, *n*=6; Figure 3h). These results further proved that necroptosis played an important role in brain injury after ICH.

### Conditioned medium from activated microglia can induce necroptosis of cultured neuron *in vitro*

**Figure 4:**
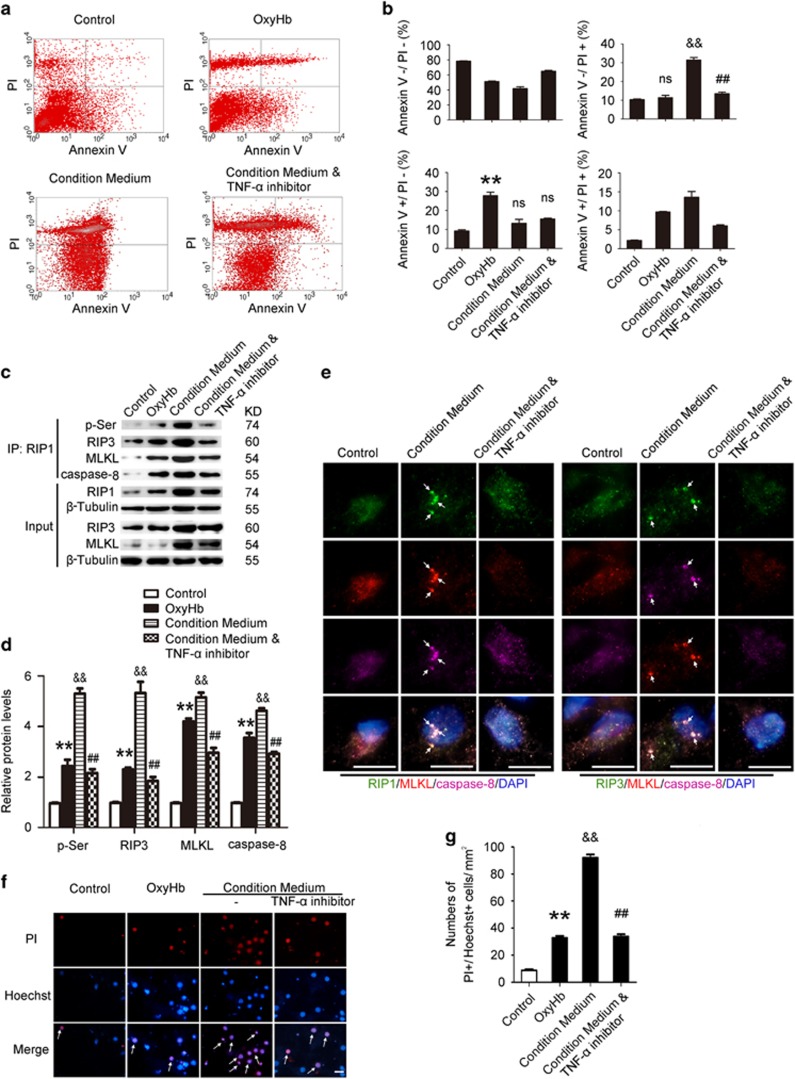
Conditioned medium-induced necroptosis in neurons *in vitro*. We used two *in vitro* ICH models to further explore the role of inflammatory factors, such as TNF-*α*, in inducing necroptosis: one is neurons treated with OxyHb directly, and the other is using supernatant of culture of microglia (stimulated with OxyHb in advance) as neurons' conditioned medium, and treated with or without inhibitor of TNF-*α*. (**a**) The necroptosis and apoptosis of neurons *in vitro* were detected by PI and Annexin V double staining and flow cytometry analysis, respectively. PI−/Annexin V− represented survival neurons, PI+/Annexin V− represented necroptotic neurons, PI−/Annexin V+ represented apoptotic neurons, and PI+/Annexin V+ represented a mixed damage of neurons. The results of flow cytometry indicated a higher ratio (~27.8%) of apoptosis and a lower ratio (~11.4%) of necroptosis when neurons were stimulated with OxyHb. However, in conditioned medium treatment group, it was a higher percentage of necroptosis (~31.5%), whereas the ratio could be significantly reduced when treated with TNF-*α* inhibitor (~13.5%). (**b**) Related bar graph showed four different conditions of neurons in various groups; NS, not significant difference (*P*=0.4642) in PI+/Annexin V− cells and **P=0.0007 in PI−/Annexin V+ cells *versus* Control group; ^&&^*P*=0.0001 in PI+/Annexin V− cells and NS, not significant difference (*P*=0.1498) in PI−/Annexin V+ cells *versus* Control group; ^##^*P*=0.0004 in PI+/Annexin V− cells and NS, not significant difference (*P*=0.3401) in PI−/Annexin V+ cells *versus* Conditioned medium group; all were unpaired *t*-test, *n*=3. (**c**) IP revealed that when treated with conditioned medium, interactions of RIP1 and RIP3, RIP1 and MLKL, and RIP1 and caspase-8 were remarkably increased. And these results were attenuated when pretreated with TNF-*α* inhibitor. (**d**) Consistent data analysis of IP. **P=0.0047 in p-Ser, **P<0.0001 in RIP3, **P<0.0001 in MLKL and **P=0.0002 in caspase-8 *versus* Control group; ^&&^*P*<0.0001 in p-Ser, ^&&^*P*=0.0006 in RIP3, ^&&^*P*<0.0001 in MLKL and ^&&^*P*<0.0001 in caspase-8 *versus* Control group; ^##^*P*=0.0003 in p-Ser, ^##^*P*=0.0018 in RIP3, ^##^*P*=0.0012 in MLKL and ^##^*P*=0.0002 in caspase-8 *versus* conditioned medium group. The mean values for the control group were normalized to 1.0, all were unpaired *t*-test, *n*=3. (**e**) Immunofluorescence analysis was performed with antibody for RIP1 /RIP3 (green), MLKL (red) and caspase-8 (purple) in cultured primary neurons under indicated treatment. Nuclei were fluorescently labeled with DAPI (blue). Representative images were shown. Arrows indicated the colocalization of RIP1-MLKL-caspase-8 and RIP3-MLKL-caspase. Scale bar=20 *μ*m. (**f**) PI and Hoechst double staining was also used in detection of necroptosis. The results showed that neurons in conditioned medium had higher ratio of necroptosis (as arrows point to, PI+/Hoechst+ cells), which could be inhibited by TNF-*α* inhibitor. Scale bar=50 *μ*m. (**g**) Numbers of PI+/Hoechst+cells. **P<0.0001 *versus* control group, ^&&^*P*<0.0001 *versus* control group, ^##^*P*<0.0001 *versus* conditioned medium group; all were unpaired *t*-test, *n*=6. All data are expressed as means±S.E.M.

To further explore the mechanism of necroptosis after ICH, we used two *in vitro* models of ICH: one is oxygen hemoglobin (OxyHb) to deal directly with the neurons, and the other is to prestimulate microglia with OxyHb, collect the supernatant as conditioned medium and then treat neurons with the conditioned medium. After these treatments, neurons were digested by trypsin into cell suspension, stained with Annexin V and PI and then detected by flow cytometry. The results of flow cytometry showed that, compared with control group, there was a higher apoptotic ratio (~27.8%) in OxyHb directly stimulated neuron group, whereas there was a higher necroptotic ratio in conditioned medium treatment group (~31.5%). In addition, Nec-1 treatment, but not z-VAD, significantly reduced the conditioned medium-induced neuron necroptosis (data not shown). However, the TNF-*α* inhibitor pretreatment significantly reduced the percentage of necroptotic neurons (~13.5% all *P*<0.01, *n*=3; Figures 4a and b).

The results of IP also demonstrated that, compared with the control, interactions of RIP1 and RIP3, RIP1 and MLKL, and RIP1 and caspase-8 were significantly increased by treatment with conditioned medium. However, interactions of RIP1 and RIP3, RIP1 and MLKL, and RIP1 and caspase-8 were remarkably inhibited by TNF-*α* inhibitor pretreatment than that in the conditioned medium group (all *P*<0.01, *n*=3; Figures 4c and d). To further clarify the interaction among RIP1-RIP3-MLKL-caspase-8, immunofluorescence staining of RIP1/MLKL/caspase-8 and RIP3/MLKL/caspase-8 was also performed on primary neurons (Figure 4e). Consistent with the results of IP, there were increased colocalizations of RIP1-MLKL-caspase-8 and RIP3-MLKL-caspase in conditioned medium-treated neurons, which were significantly inhibited by TNF-*α* inhibitor. PI and Hoechst staining also showed that treatment with conditioned medium increased the ratio of necroptosis in neurons, but this can be inhibited by TNF-*α* inhibitor pretreatment (both *P*<0.0001, *n*=6; Figures 4f and g). These results suggested that TNF-*α* in conditioned medium may be an important factor of inducing necroptosis in neurons.

### Phosphorylation of RIP1 has an essential role in activation of necroptosis in neuron *in vitro*

**Figure 5:**
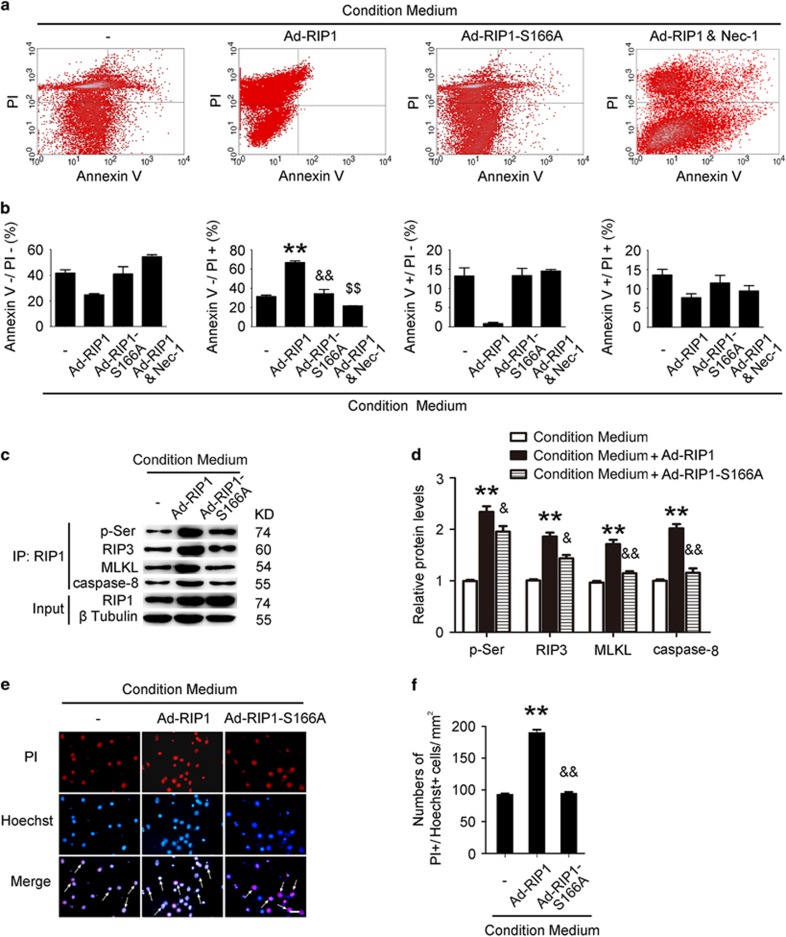
Phosphorylation of RIP1 switched necroptosis in cultured neurons in model of ICH *in vitro*. As phosphorylation of RIP1 has an essential role of necroptosis, we used overexpression and with a mutation of phosphorylation site (S166A) of RIP1 to investigate its role in necroptosis in ICH. (**a**) Flow cytometry indicated that, compared with the conditioned medium group, it turned up a higher ratio of necroptosis (~66.7%) and a lower ratio of apoptosis (~0.9%) in neurons during overexpression of RIP1 by Ad-RIP1 transfection. Besides, this phenomenon could be significantly reduced in the S166A group (necroptosis ~34.2%) and in the Nec-1 treatment group (necroptosis ~21.7%). (**b**) Related bar graph showed four different conditions of neurons that had been stated above. **P=0.0001 in PI+/Annexin V− cells *versus* the conditioned medium group; ^&&^*P*=0.0027 in PI+/Annexin V− cells *versus* the Ad-RIP1 group; ^$$^*P*<0.0001 in PI+/Annexin V− cells and ^$$^*P*<0.0001 in PI−/Annexin V+ cells *versus* the Ad-RIP1 group; all were unpaired t-test, *n*=3. (**c**) IP demonstrated that interactions of RIP1 and RIP3, RIP1 and MLKL, and RIP1 and caspase-8 were increased in the Ad-RIP1 group. These increased interactions could be attenuated remarkably in the S166A group. (**d**) Consistent data analysis of IP. **P=0.0015 in p-Ser, **P=0.0004 in RIP3, **P=0.0013 in MLKL and **P=0.0003 in caspase-8 *versus* the conditioned medium group; ^&^*P*=0.0493 in p-Ser, ^&^*P*=0.0126 in RIP3, ^&&^*P*=0.0036 in MLKL and ^&&^*P*=0.0018 in caspase-8 *versus* the Ad-RIP1 group. The mean values for the control group were normalized to 1.0; all were unpaired *t*-test, *n*=3. (**e**) PI and Hoechst staining indicated that the ratio of necroptosis upregulated (as arrows point to, PI+/Hoechst+ cells) in the overexpression group, whereas mutation of the phosphorylation site inhibited this effect. Scale bar=50 *μ*m. (**f**) Numbers of PI+/Hoechst+ cells, **P<0.0001 *versus* the conditioned medium group; ^&&^*P*<0.0001 *versus* Ad-RIP1 group; all were unpaired *t*-test, *n*=6. All data are expressed as means±S.E.M.

Phosphorylation of RIP1 is an essential element of activation of necroptosis.^15^ We detected the phosphorylation of RIP1 by IP using anti-RIP1 antibody followed by immunoblotting for anti-phosphorylation serine (p-Ser) antibody. The results of IP suggested that the phosphorylation (serine site) of RIP1 was obviously increased in the conditioned medium treatment group and could be inhibited by TNF-*α* inhibitor pretreatment (both *P*<0.01, *n*=3; Figures 4c and d). To further explore the molecular mechanism of RIP1 in necroptosis after ICH, we used overexpression with a mutation of phosphorylation site (S166A) of RIP1. After transfection and being cultured for another 24 h, neurons were digested by trypsin into cell suspension, stained with Annexin V and PI and then detected by flow cytometry. The results of flow cytometry revealed that upregulating the expression of RIP1 could increase the percentage of necroptosis (~66.7%) in neurons, but overexpression of mutation of phosphorylation site (S166A) of RIP1 has no obvious effect in inducing necroptosis of neurons (~34.2%), and Nec-1 treatment can also inhibit the necroptosis induced by RIP1 overexpression (~21.7%, all *P*<0.01, *n*=3; Figures 5a and b).

The results of IP also demonstrated that, compared with the control, interactions of RIP1 and RIP3, RIP1 and MLKL, and RIP1 and caspase-8 were significantly increased by overexpression of RIP1. However, interactions of RIP1 and RIP3, RIP1 and MLKL, and RIP1 and caspase-8 were remarkably inhibited by mutation of phosphorylation site (S166A) of RIP1 than those in the overexpression of wild type of the RIP1 group (all *P*<0.01, *n*=3; Figures 5c and d). PI and Hoechst staining also showed that overexpression of wild-type RIP1 increases the ratio of necroptosis in neurons, but this effect can be inhibited by mutation of the phosphorylation site (S166A) of RIP1 (all *P*<0.0001, *n*=6; Figures 5e and f). These results all suggested that phosphorylation in the 166th site of RIP1 may be an important element of inducing necroptosis in neurons.

## Discussion

Cell death is the important reason leading to brain injury after ICH. Almost previous studies believed that the form of cell death is apoptosis in brain tissues after ICH; few researches put attention to the role of necrosis. Initiation factors of apoptosis include downregulation of blood flow and energy metabolism around the hematoma; a variety of enzymes that are activated in the blood after ICH activate the apoptosis signal, and mechanical damage of hematoma directly causes apoptosis.^5, 16, 17^ Apoptosis theory can partly explain the mechanism of cell death in brain tissues after ICH. However, recent study found that the dead brain cells release a series of proteins from the cytoplasm after ICH; these proteins were known as danger-associated molecular patterns. The most typical representative is the high-mobility group protein 1, which can stimulate the inflammatory response that aggravates secondary brain injury after ICH.^18^ These results pose challenge to apoptosis theory in ICH; scholars widely recognized that cell apoptotic process and released apoptotic bodies do not cause inflammation.^15^ Thus, presumably, another form of cell death in addition to apoptosis might exist in brain tissues and can stimulate inflammatory response after ICH. On the other hand, microglia were rapidly activated after ICH and released substantial inflammatory factors (such as TNF-*α*); previous studies have confirmed that TNF-*α* can not only induce apoptosis but also necroptosis; so whether necroptosis exists in brain tissues after ICH becomes a question.

Our present study showed that conditioned medium, especially the key composition TNF-*α*, from activated microglia induced necrosis of neurons after ICH for the first time. We found that necroptosis existed in brain tissues after ICH, and it had an important role in neuronal dysfunction, brain edema and BBB permeability after ICH. RIP1 inhibitor Nec-1 can remarkably attenuate neurological dysfunction, brain edema and BBB injury, and combinational use with apoptosis inhibitor z-VAD has a better effect, suggesting that necroptotic pathway and RIP1 may become targets for the treatment of brain injury after ICH. We also observed that treatment of ICH rats with Nec-1 does not affect the apoptosis of brain cells, and the use of z-VAD treatment of ICH rats also does not affect the necroptosis in brain tissues; these results indicated that apoptosis and necroptosis were coexisting and were relatively independent after ICH. Nec-1 and z-VAD could inhibit apoptosis and necroptosis, respectively, whereas combinational treatment of Nec-1 and z-VAD was more effective in treating ICH than each inhibitor alone. In addition, there may be a crosstalk between apoptosis and necroptosis in the progression of ICH, which needs further investigation. In addition, while our research was in progress, Su *et al.*^19^ reported that Nec-1 ameliorated brain injury after ICH in a collagenase-induced ICH model in mice.^19^ A recent report also showed that Nec-1 reduced neurovascular injury after ICH in a collagenase-induced ICH model in mice.^20^ As our present study showed that, TNF-*α*, an important inflammatory cytokine, may be a key factor in neurons' necrosis after ICH, the side effects of collagen on inflammatory response should not be ignored in necrosis-related studies. In this study, we researched neurons' necrosis in autologous blood injection ICH model for the first time.

In many previous studies, stimulation of neurons with OxyHb was an *in vitro* model of ICH.^21^ However, the inflammation is an important event in second brain injury after ICH; activated microglia released an amount of inflammatory cytokines such as TNF-*α* and IL-1*β*, which promote brain injury after ICH.^22^ Thus, in this study, we used OxyHb-stimulated microglia and collected the supernatant as conditioned medium, and then used conditioned medium to treat the neurons, as an ICH model *in vitro*. This model highlights the stimulation of inflammatory factor on neurons, and results of ELISA also suggested that the level of TNF-*α* was significantly increased in conditioned medium compared with the medium of nontreated microglia (data not shown). We found that necroptotic rate of neurons in this model was significantly higher than that in the OxyHb-treated neurons, suggesting that inflammatory factors released by activated microglia are a key factor leading to cell necroptosis in brain tissues. A previous study reported that, in a murine model of ICH, postinjury treatment with the TNF-*α* antibody resulted in less neuroinflammation and reduction in functional deficit;^23^ combining with our results, we can conclude that ameliorating brain injury after ICH by blocking TNF-*α* is partially because of reducing the necroptosis in brain tissues. Our results also suggested that multiple stimuli factors coexisted in brain tissues after ICH, and led to cell death by different pathways; the dominant stimulus in local microenvironment might be the main reason leading to brain cell death. At the same time, our results demonstrated that use of multiple *in vitro* models may be more appropriate in the study of brain injury following ICH.

Unlike apoptosis, the release of cell contents will cause inflammation after the cell necroptosis. Interestingly, an important initial factor of necroptosis is stimulation of inflammatory factor (such as TNF-*α*), and necroptosis also can further promote the inflammation, these suggested that possibly have a positive feedback relationship between necroptosis and inflammation in brain injury after ICH. The effect of this positive feedback relationship in the pathophysiological processes after ICH needs to be further confirmed. There are also some deficiencies in this study; although the role of necroptosis in the brain injury following ICH was explored, the molecular mechanism is still uncertain. On the other hand, there are many kinds of inflammatory cytokines released by activated microglia, such as IL-1*β*, IL-6 and IL-18; whether these inflammatory factors also can cause cell necroptosis in brain tissues after ICH is not clear.

**Figure 6:**
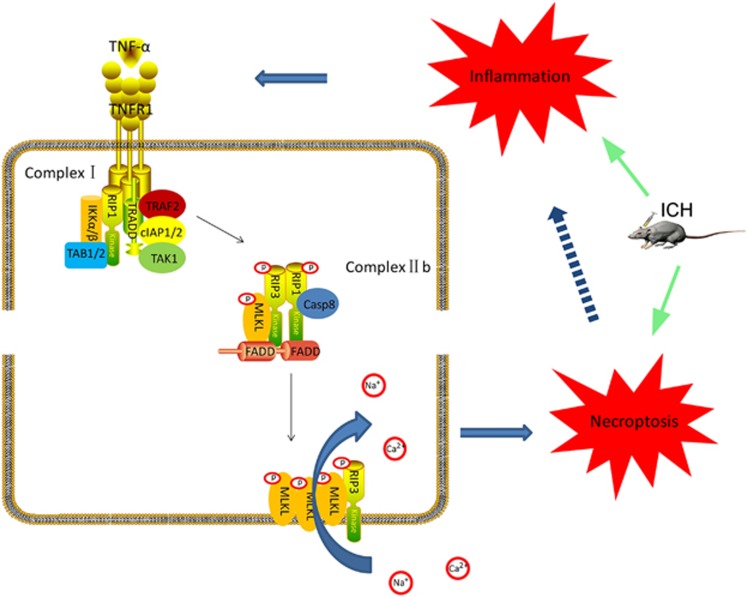
Hypothesized model for molecular mechanism involved in necroptosis in brain tissues after ICH. After ICH occurs, microglia were rapidly activated and released an amount of inflammatory cytokines such as TNF-*α*. TNFR1 can spontaneously trimerize at the plasma membrane. When TNF-*α* binds to TNFR1, the conformation of these receptor trimers would be changed, allowing their cytosolic tails recruit multiple proteins, and then develop a complex (complex I) including TRADD (TNF-*α* receptor-associated death domain), RIP1, TRAF2 (TNFR associated factor 2), cIAP1 and cIAP2 (cellular inhibitor of apoptosis 1/2). Deubiquitylation of RIP1 mediates its transition from complex I to complex II, and then cooperates with RIP3 for recruitment of MLKL (mixed lineage kinase domain-like protein), FADD (FAS-associated protein with a death domain) and caspase-8. In this complex IIb, RIP1 and RIP3 are inhibited by caspase-8. When caspase-8 is inactive, complex IIb would carry out the TNF-*α*-mediated necroptotic pathway. Further, complex of MLKL and RIP3 transfers to the cell membrane and forms a channel, and then the internal flow of Ca^2+^ or Na^+^ is caused. Finally, the cell is dead by the necroptotic pathway. As necroptosis can further promote inflammation, it suggests that it can possibly have a positive feedback relationship between necroptosis and inflammation in brain injury after ICH.

In summary, our study confirmed that the RIP1-mediated necroptosis exists in brain tissues, and it has an important role in brain injury following ICH; on the other hand, we used the *in vitro* model of ICH suggesting that the release of TNF-*α* from activated microglia might be an important factor inducing necroptosis in ICH (Figure 6). These findings further revealed the causes of cell death and the relationship between cell death and inflammation in brain injury after ICH and provide a potential therapeutic target for secondary brain injury after ICH.

## Materials and Methods

### Animals

Adult male Sprague–Dawley (SD) rats, weighing ~300 g, were provided by the Animal Center of Chinese Academy of Sciences (Shanghai, China). Experimental protocols were approved by the Animal Care and Use Committee of Soochow University, and were implemented with reference to the National Institutes of Health guidelines. The animals were freely fed and housed in a quiet environment (indoor temperature of ~18–22 °C). Additionally, we strived as much as possible to minimize the number of animals that were used and reduce their suffering. Besides, primary neuronal and microglial cultures (*in vitro*) were prepared using 16–18-day-old pregnant SD rats.

### Experimental design

**Figure 7:**
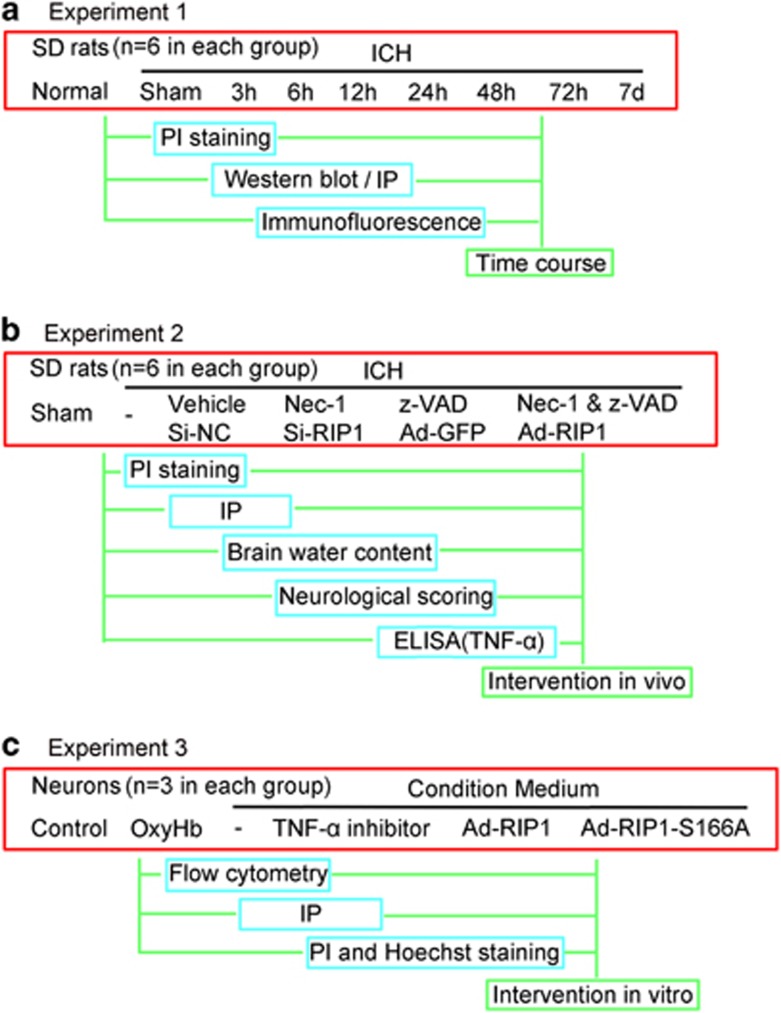
Experimental designs. (**a**) Experiment 1 was designed to show the time course of RIP1 expression after ICH and to determine a time point for the next experiment. (**b**) Experiment 2 was designed to explore the roles of RIP1 and necroptosis in brain injury after ICH *in vivo*. (**d**) Experiment 3 was designed to study the potential mechanism of RIP1 and necroptosis in brain injury after ICH *in vitro.*

In experiment 1, 54 rats (70 rats were used, 54 rats survived after the surgery) were randomly assigned to nine groups of six rats each; the normal group, the Sham group and seven experimental groups were arranged by time – 3, 6, 12, 24, 48, 72 h and 7 days after ICH. Arriving at separate time points after SAH, all rats were killed and cerebral tissue samples were collected for analysis (Figure 7a). In experiment 2, 60 rats (75 rats were used, 60 rats survived after the surgery) were randomly divided into 10 groups – the Sham group, the ICH group, the ICH+vehicle group, the ICH+Nec-1 group, the ICH+z-VAD group, the ICH+Nec-1+z-VAD group, the ICH+Si-negative control group, the ICH+SiRNA-RIP1 group, the ICH+Ad-GFP group and the ICH+Ad-RIP1 group. At 24 h after ICH, all rats were examined for behavioral impairment, and then brain samples were collected (Figure 7b). In experiment 3, primary cultured neurons were used and partitioned into six groups – the control group, the OxyHb group, the conditioned medium group, the conditioned medium+TNF-*α* inhibitor group, the conditioned medium+Ad-RIP1 group and the conditioned medium+Ad-RIP1-S166A group (Figure 7c). Detailed information about each group was shown in special procedures as below.

### Establishment of ICH model

ICH model *in vivo* was established by injection of autologous blood.^24^ After anesthesia with intraperitoneal injection of 4% chloral hydrate at a dosage of 1 ml/100 g, 100 *μ*l of autologous blood was collected from the heart, and then the rats were fixed in the stereotaxic frame (Zhenghua Biological Equipment Co. Ltd, Anhui, China). The scalp was exposed and made drilling a hole corresponding to right basal ganglia (0.2 mm anterior to the intersection between the coronal suture and sagittal midline and 3.5 mm to the right of the sagittal suture). A microsyringe was affixed to the stereotactic frame and a needle was slowly inserted (5.5 mm in depth), and then 100 *μ*l of autologous blood was slowly injected (20 *μ*l/min). Before slowly withdrawing the needle, it was kept in place for another 5 min. Rats in the Sham group were intracerebrally injected with 100 *μ*l physiological saline solution. The bone hole was sealed with bone wax, and skin incision was disinfected and sutured. During the establishment of the model, rats' vital signs were monitored and maintained in normal level.

### Primary neuron- and microglia-enriched cultures

Neuron-enriched cultures were prepared from brains of fetal rats (from 16–18-day-old pregnant SD rats).^25^ The meninges and blood vessels were removed from the brain and then brain tissues were digested with 0.25% trypsin (with EDTA) for 5 min at 37 °C. The tissues were washed three times with PBS to terminate trypsin digestion. Then, brain tissue suspensions were centrifuged at 1500 r.p.m. for 5 min, and the cells were suspended in a Neurobasal-A medium containing 2% B27, 2 mM l-glutamine, 50 U/ml penicillin and 50 U/ml streptomycin (all from Gibco, Carlsbad, CA, USA). Finally, cells were plated in 6- or 12-well plates in a fresh medium and later half the medium was changed with fresh medium every 2 days.

For primary microglial cultures, the whole brains of 1- day-old rats were used.^26^ The digestion was similar to neurons, but the pellets after centrifugation were suspended in DMEM/F12 containing 10% fetal bovine serum, 1 mM sodium pyruvate, 2 mM l-glutamine, 100 mM nonessential amino acids, 50 U/ml penicillin and 50 U/ml streptomycin (all from Gibco). Then, cells were seeded into 150 cm^2^ culture flask in a fresh medium and half the medium was changed with fresh medium every 2 days. Two weeks after initial seeding, a confluent polylayer of glial cells could be observed. The microglia was separated from astrocytes by shaking the flask at 150 r.p.m. for 4 h, and then it was collected by centrifugation and reseeded in 12-well plates with fresh medium.

### Drug administration

Nec-1 was prepared in DMSO at a concentration of 1 *μ*g/3 *μ*l,^27^ and z-VAD (all from Santa Cruz Biotechnology, Santa Cruz, CA, USA) was prepared in DMSO at a concentration of 100 *μ*M.^10^ At 1–2 h before ICH, both the inhibitors were injected into the lateral cerebral ventricle at a volume of 3 *μ*l. Equal volumes of DMSO were used as vehicle. For *in vitro* experiments, the TNF-*α* inhibitor (Santa Cruz Biotechnology) was also dissolved in DMSO at a final concentration of 50 *μ*M in a neuronal medium. The final concentration of Nec-1 and z-VAD was 30 and 100 *μ*M, respectively, in a neuronal medium.

### ICH models *in vitro*

An ICH model *in vitro* was established by neuronal stimulation using OxyHb.^21^ Neurons were treated with OxyHb (10 *μ*M) for 6 h at 37 °C in 5% CO_2_, and then cell medium was removed, washed three times with PBS and followed by other experiments.

The other *in vitro* ICH model used conditioned medium to treat neurons. First, microglia were treated with OxyHb (10 *μ*M) and then incubated for 24 h at 37 °C with 5% CO_2_. The supernatant collected was centrifuged at 10 000 r.p.m. for 5 min and then was transferred to a conditioned medium. Half of the neuronal medium was changed with conditioned medium and incubated for another 6 h at 37 °C with 5% CO_2_. The cell medium was removed, washed three times with PBS and followed by other experiments.

### Transfection of siRNA and adenoviruses *in vivo* and *in vitro*

The following three kinds of recombinant adenoviruses were used: (1) adenoviruses containing rat RIP1 (Ad-RIP1; Genbank ID: 157824040) that was used to overexpress RIP1 protein; (2) adenovirus with rat RIP1 with mutation of S166A (Ser166 of RIP1 was mutated to alanine, Ad-RIP1-S166A); and (3) adenovirus with human GFP (Ad-GFP) as a control to Ad-RIP1. Ad-RIP1 and Ad-RIP1-S166A (both 6 × 10^9^ PFU/ml) and Ad-GFP (2 × 10^9^ PFU/ml) were produced by Genescript (Nanjing, China). All of them were stored at −80 °C and diluted to 1 × 10^9^ PFU/ml in an enhanced transfection solution (Genescript) before intracerebroventricular injection *in vivo* and diluted to 1 × 10^8^ PFU/ml before being transfected to the cultured neuron.

The following two kinds of siRNAs were used: (1) disorganizing rat RIP1 mRNA (Si-RIP1) to silence its transcription and (2) scramble siRNA (Si-negative control) (both from Genescript). According to the manufacturer's instructions for Entranster-*in vivo* RNA transfection reagent (Engreen, Shanghai, China), 500 pmol RIP1 siRNA and 500 pmol scramble siRNA were dissolved in 5 *μ*l RNase-free water. Then, 10 *μ*l Entranster-*in vivo* RNA transfection reagent was added to 5 *μ*l siRNA or 5 *μ*l scramble siRNA. After mixing for another 15 min, Entranster-*in vivo*-siRNA mixture was injected intracerebroventricularly 24 h before ICH. RIP1 siRNA sequences were as follows: sense, 5′-GGAACAACGGAGUAUAUAAdTdT-3′ and Antisense: 3′-dTdTCCUUGUUGCCUCAUAUAUU-5′.

### Western blot analysis

Western blot analysis was performed as indicated previously.^25^ Briefly, the brain samples or extracted cells were mechanically lysed in RIPA lysis buffer (Beyotime, Shanghai, China). Then we used enhanced BCA Protein Assay Kit (Beyotime) to measure protein concentrations by the bicinchoninic acid method. The protein samples (50 *μ*g per lane) were then loaded onto a 10% SDS-polyacrylamide gel, separated and electrophoretically transferred to a polyvinylidene difluoride membrane (Millipore Corporation, Billerica, MA, USA), which was then blocked with 5% bovine serum albumin (BioSharp, Anhui, China) (1 h at room temperature). Then, the membrane was incubated for 12 h at 4 °C with primary antibodies. The primary antibodies used were RIP1, RIP3, MLKL, caspase-8, p-Ser (all from Santa Cruz Biotechnology) and albumin (Abcam, Cambridge, UK; ab106582). Besides, the *β*-tubulin was also detected and served as a loading control. Later, the membrane was incubated with related HRP-conjugated secondary antibody (Santa Cruz Biotechnology) for 2 h at room temperature. We revealed the band signals via the Enhanced Chemiluminescence (ECL) Kit (Beyotime), and the relative quantity of proteins was analyzed via the Image J Software (NIH, Bethesda, MD, USA) and normalized to that of the loading control as discussed previously. In addition, the levels of phosphorylation were evaluated as the ratio of phosphoprotein to total protein.

### Immunoprecipitation

Briefly, according to our previous report,^25^ the brain samples were lysed in ice-cold RIPA lysis buffer (Beyotime). For IP, the lysate was incubated with specific antibodies against RIP1 or rabbit IgG (negative control) overnight at 4 °C with agitation. Protein A+G Sepharose beads were then added to each immune complex, and the lysate–bead mixture was incubated for 4 h at 4 °C under rotary agitation. SDS-PAGE and immunoblotting were then performed for further protein separation and detection.

### Immunofluorescent staining

The brain samples were fixed in 4% paraformaldehyde, embedded in paraffin and then cut into 4 *μ*m sections. The cultured neurons were fixed in 4% paraformaldehyde. Then, the sections and neurons were incubated with primary antibody against RIP1, RIP3, MLKL and caspase-8 and secondary antibodies. Normal rabbit IgG, normal mouse IgG and normal goat IgG were used as negative controls for the immunofluorescence assay (data not shown). Nuclei were stained with DAPI mounting medium. Finally, the sections and neurons were observed in a fluorescence microscope (OLYMPUS BX50/BX-FLA/DP70; Olympus Co., Tokyo, Japan). The relative fluorescence intensity was analyzed with the Image J program. The quantitative analysis was performed by an observer who was blinded to the experimental group.

### PI and TUNEL staining

For all experiments, PI (Sigma-Aldrich, St. Louis, MO, USA) was administered intraperitoneally (1 *μ*g/g) while the ICH model was established.^28^ According to the manufacturer's protocol (Roche, Mannheim, Germany), we detected cell apoptosis via TUNEL staining. The brain was removed and frozen in liquid nitrogen and then stored at −80 °C. Then, brain sections (12 *μ*m) were cut on a cryostat 150 to 200 *μ*m apart along the anterior–posterior lesion and were placed on poly-l-lysine-coated glass slides (−80 °C). Next, the sections were incubated with TUNEL staining (37 °C for 1 h). After washing three times with PBST, sections were visualized by a fluorescence microscope (OLYMPUS BX50/BX-FLA/DP70; Olympus Co.). In conclusion, PI/TUNEL+ cells were counted by observers blinded to the groups of experiment. To evaluate necropoptosis/apoptosis of cells, we examined and photographed six microscopic fields per sample and the index was defined as the average number in each section. As reported previously, PI+/TUNEL− is defined as the pure necropoptosis, PI−/TUNEL+ is defined as pure apoptosis, and PI+/TUNEL+ is defined as the mixed cell death.^29^

### Brain edema and BBB injury

As described previously,^21^ the wet–dry method was adopted to evaluate the index of brain edema and BBB injury. Briefly, after brain tissues were removed and collected, the samples were weighed immediately (wet weight), dried (100 °C for 72 h) and then reweighed (dry weight). Thus, brain edema was calculated as ((wet weight−dry weight)/wet weight) × 100%. Meanwhile, the level of albumin in brain tissues was regarded as the index of BBB injury. The western blot was used to test the level of albumin.

### Neurobehavioral evaluation

At 24 h after ICH, all the rats in the experiments were examined for behavioral impairment using a scoring system and were monitored for appetite, activity and neurological defects, as reported previously^30^ (for more details see Table 2).

### Assay of inflammatory cytokines (TNF-*α*)

We used specific Rat Tumor necrosis factor α, TNF-α ELISA KIT (Bio-Swamp; Hubei, China) to quantify the levels of TNF-*α* in the CSF according to the manufacturer's instructions.

### Annexin V and PI staining *in vitro*

After various treatments, neurons were trypsinized by 0.25% trypsin (without EDTA) and centrifuged at 1500 r.p.m. for 5 min, and the resulting cell pellet was resuspended in 500 *μ*l binding buffer. Later, 5 *μ*l Annexin V and 5 *μ*l PI (Beyotime, Shanghai, China) were added to the cell suspension. After 20 min of incubation at 37 °C in the dark, the cells were analyzed by flow cytometry (FACS Cabibur; BD, San Diego, CA, USA) and at least 20 000 events per sample were recorded.

### PI and Hoechst staining *in vitro*

After various treatments, add 5 *μ*l Hoechst and 5 *μ*l PI (Beyotime, Shanghai, China) to the cell medium. After 20 min of incubation at 4 °C in the dark, the cells were analyzed by a fluorescence microscope (OLYMPUS BX50/BX-FLA/DP70; Olympus Co., Tokyo, Japan.)

### Statistical analysis

All data are presented as mean±S.E.M. GraphPad Prism 5.0 software (GraphPad, San Diego, CA, USA) was used for statistical analysis. Data sets were tested for normality of distribution with Kolmogorov–Smirnov test. Data groups (two groups) with normal distribution were compared using two-sided unpaired Student's *t*-test, and the Mann–Whitney *U*-test was used for nonparametric data. *P*<0.05 indicates a statistically significant difference.

## Figures and Tables

**Table 1:** Clinical behavior scores in each group (*n*=6)

**Group**	**Score (mean±S.E.M.)**
Normal	0.17±0.17
Sham	0.33±0.21
ICH	2.67±0.33^a^
ICH+vehicle	3.17±0.31^b^
ICH+Nec-1	1.67±0.21^c^
ICH+z-VAD	1.83±0.17^d^
ICH+Nec-1 and z-VAD	1.17±0.17^e^
ICH+Si-NC	2.67±0.21^f^
ICH+Si-RIP1	1.67±0.21^g^
ICH+Ad-GFP	3.00±0.37^h^
ICH+Ad-RIP1	4.33±0.21^i^

Abbreviations: Ad-GFP, adenovirus with GFP; Ad-RIP1, adenovirus with RIP1; ICH, intracerebral hemorrhage; Nec-1, necrostatin-1; RIP1, receptor-interacting protein 1; Si-NC, Si-negative control.

a *P*=0.0043 *versus* Sham group (Mann–Whitney test).

b *P*=0.2959 *versus* ICH group (unpaired *t*-test).

c *P*=0.0094 *versus* ICH+vehicle group (Mann–Whitney test).

d *P*=0.0096 *versus* ICH+vehicle group (Mann–Whitney test).

e *P*=0.0341 *versus* ICH+z-VAD group (Mann–Whitney test).

f *P*=0.9290 *versus* ICH group (Mann–Whitney test).

g *P*=0.0185 *versus* ICH+Si-NC group (Mann–Whitney test).

h *P*=0.5155 *versus* ICH group (unpaired *t*-test).

i *P*=0.0205 *versus* ICH+Ad-GFP group (Mann–Whitney test).

**Table 2:** Neurobehavioral evaluation

**Category**	**Behavior**	**Score**
Appetite	Finished meal	0
	Left meal unfinished	1
	Scarcely ate	2
		
Activity	Walk and reach at least three corners of the cage	0
	Walk with some stimulations	1
	Almost always lying down	2
		
Deficits	No deficits	0
	Unstable walk	1
	Impossible to walk	2
